# Risk Assessment for the Population of Kyiv, Ukraine as a Result of Atmospheric Air Pollution

**DOI:** 10.5696/2156-9614-10.25.200303

**Published:** 2020-01-22

**Authors:** Oleksandr Popov, Andrii Iatsyshyn, Valeriia Kovach, Volodymyr Artemchuk, Iryna Kameneva, Dmytro Taraduda, Vitaliy Sobyna, Dmitry Sokolov, Maksim Dement, Teodoziia Yatsyshyn

**Affiliations:** 1 State Institution, Institute of Environmental Geochemistry of National Academy of Sciences of Ukraine, Kiev, Ukraine; 2 Pukhov Institute for Modelling in Energy Engineering of National Academy of Sciences of Ukraine, Kiev, Ukraine; 3 National University of Civil Defense of Ukraine, Kharkiv, Ukraine; 4 Ivano-Frankivsk National Technical University of Oil and Gas, Ivano-Frankivsk, Ukraine

**Keywords:** air pollution, air quality, risk assessment, population health

## Abstract

**Background.:**

According to the World Health Organization, 92% of the world's population lives in places where air quality levels exceed recommended limits. Recently, Ukraine had the most deaths per every 100,000 people (out of 120 countries) attributed to atmospheric air pollution. High levels of atmospheric air pollution have been observed not only in typically industrial regions, but in Ukraine's capital, Kyiv, as well.

**Objectives.:**

The aim of the present study was to establish the state of air pollution in Kyiv and perform a risk assessment of associated human health effects.

**Methods.:**

Using official statistics and state monitoring data, the study aimed to identify and analyze risks to the health of Kyiv's population associated with air pollution. The following methods were used: systematic, functional and comparative analysis, risk theory, mathematical modeling, probability theory and mathematical statistics, as well as geographic information system technologies for digital map design and objective-oriented methodology for software design systems.

**Results.:**

The risk values across different areas of the city varied significantly, indicating that atmospheric air quality remains unstable. Areas with the highest and lowest risk values were identified.

**Conclusions.:**

The environmental state of atmospheric air in Kyiv requires greater attention and additional research to identify the causes of air pollution, along with implementation of measures to improve air quality.

**Competing Interests.:**

The authors declare no competing financial interests.

## Introduction

Air pollution is a large problem in many countries around the world. The main sources of atmospheric air pollution are transportation, power companies, chemically hazardous enterprises, metallurgy, machine-building, metal-processing enterprises and industries, building materials industries, and lumber operations, among others.^1^ Atmospheric pollution is considered to be the most dangerous form of pollution by volume, as pollution of industrially developed cities by toxic agents becomes irreversible and has negative impacts on the health of the population.

According to the World Health Organization (WHO), 92% of world population breathes air polluted beyond the acceptable limits.^2^ Ukraine is no exception and recently (in September, 2017) ranked first (out of 120 world countries) in deaths caused by atmosphere air pollution, per 100,000 people. Air pollution in Ukraine causes four times more deaths per capita than the five cleanest countries altogether, according to the WHO.^3^

The main sources of pollution in Kyiv are motor transport and energy enterprises (thermal stations), enterprises of the construction industry, mechanical engineering, chemical-pharmaceutical, and the food industry. In 2015, the total amount of pollutants in the atmosphere from stationary and mobile sources in Kyiv amounted to 144.3 thousand tons. More detailed data on emissions from stationary technogenic facilities in Kiev are presented in Table 1.^4^

**Table 1:** Dynamics of Emissions from Stationary Sources

**Stationary sources**	**2010**	**2013**	**2014**	**2015**	**2016**
Total air emissions from stationary pollution sources, (thousand tons)	28.6	31.9	31.4	26.7	34.3
Air emissions from stationary pollution sources per km^2^, (tons)	34.3	38.1	37.6	31.9	41.1
Air emissions from stationary pollution sources per capita, (kg)	10.3	11.2	10.9	9.2	11.8
Air emissions of particulate matters from stationary pollution sources, (thousand tons)	4.0	4.8	4.9	4.7	5.8
Air emissions of sulfur dioxide from stationary pollution sources, (thousand tons)	7.6	11.3	9.9	6.5	12.3
Air emissions of nitrogen dioxide from stationary pollution sources, (thousand tons)	9.8	9.1	7.8	6.8	7.5
Air emissions of carbon monoxide from stationary pollution sources, (thousand tons)	2.8	2.5	2.1	2.1	2.2
Air emissions of non-methane volatile organic compounds from stationary pollution sources, (thousand tons)	3.5	3.4	5.9	5.8	5.7

The air monitoring system in Ukraine is currently ineffective and has significant problems. There is no data collection and analysis center and monitoring results are stored in paper form. Most atmospheric scientific research results with some elements of statistical and comparative analysis do not consider the environmental risks to public health. Therefore, a risk assessment of the health of Kyiv's population due to air pollution supported by data and analysis is urgently needed. The aim of the present study was to establish the state of air pollution in Kyiv and perform a risk assessment of associated human health effects.

## Methods

Kyiv, the capital of Ukraine, is one of the biggest and oldest cities in Europe. Kyiv is situated in the south of Ukraine, on the border of Polissya. The Dnipro River runs through the city and there is forest steppe on either side of the river. Kyiv has an area of 836 km^2^. The climate is moderately continental, with mild winters and warm summers. The average monthly temperature in January and July is −3.5°C and +20.5°C, respectively, with an average annual precipitation of 649 mm. Forests, parks and gardens make up more than half of the almost 80 000 hectares. The population in Kyiv was 2.9 million in December 2017, according to data of the General Statistics Department.^5^ The present study was conducted from September 2016 till September 2018.

**Figure 1:**
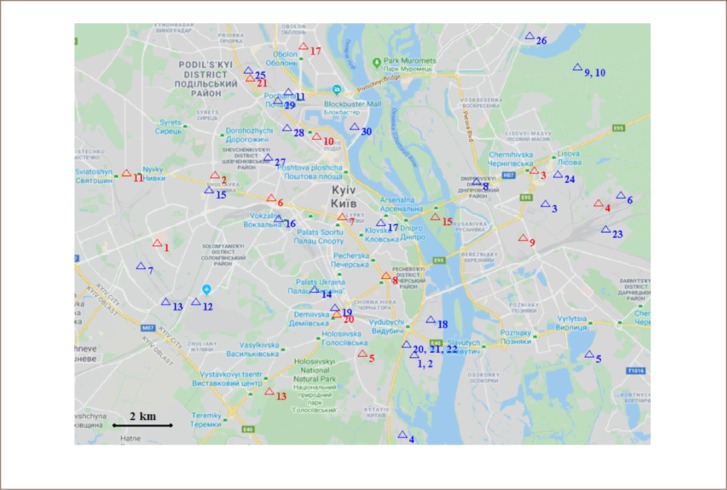
Primary enterprises impacting air quality in Kyiv (blue triangles, see Table 6) and atmospheric air pollution observation stations (red triangles)

In Ukraine, programs monitoring the quality of atmospheric air are aimed at the control of primary pollutants at stationary and mobile stations. In Kyiv, the Central Geophysical Observatory systematically monitors the content of pollutants in atmospheric air at 16 stationary (but manual) pollution observation stations (POS) with samples taken every 6 days, 3–4 times a day. These stations collect data on suspended substances, sulfur dioxide, carbonic oxide, hydrogen sulfide, phenol, hydrogen fluoride, ammonia, formaldehyde, and eight heavy metals. Their composition is defined depending on the emissions of pollutants in atmospheric air from enterprises located in the area of the observation stations. The distribution of POS in Kyiv is shown in Figure 1.

**Abbreviations**
*MPC*: Maximum permissible concentration
*PJSC*: Public joint stock company
*POS*: Pollution observation stations
*PrJSC*: Private joint stock company
*RCI*: Risk of chronic intoxication
*RITE*: Risk of immediate toxic effects
*SMC*: Software-modeling complex

In the countries of the former USSR, health standards for permissible pollutants burden consists of maximum permissible concentration (MPC), relatively safe impact levels and different complex values. In Ukraine, and most post-Soviet states, there are approved MPCs for pollutants used for the assessment of atmosphere air. Maximum permissible concentrations are set for each substance and for certain combinations of pollutants in atmospheric air. Maximum permissible concentrations of pollutants in the atmospheric air of populated areas are regulated as daily averages and maximum single MPC.

When the concentration values are taken, they are compared to maximum single values of those concentrations (thus the amount of excess is defined).

The average monthly or yearly values are likewise compared with MPCs of daily average values.

In order to assess atmospheric air pollution levels, the air pollution index was used, following Equation 1.

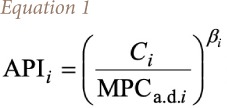
where, API is the air pollution index; C_i_ is the average concentration of *i*-additive in time (per month or per year) calculated for the station, city or a group of cities; MPC_a.d.*i*_ is the maximum permissible daily average concentration of a pollutant in the air; and *β*_*i*_ is the dimensionless constant of reducing the damage of *i*-substance to the damage of sulfur dioxide which accepts the values of 1.7; 1.3; 1.0; 0.9 for 1, 2, 3, 4 classes of hazard of pollutants, respectively.

The above-mentioned health standards characterize only the level of chemical air pollution of a territory under investigation and do not define the impact of pollution on human health. Therefore, a risk assessment was conducted to determine the probability of negative effects on human health for individuals or groups of people affected by a particular chemical reagent. It is characterized by the value that lies within (0…1) interval, where 0 indicates the absence of effect and 1 indicates the greatest effect.

In order to assess the negative impact of anthropogenic air pollution on the health of a population which may manifest itself in immediate or chronic toxic effects (carcinogenic and immunotoxic), two groups of models have been introduced: threshold and non-threshold.^6^ Acute toxicity (immediate toxic manifestations) has a definite threshold character. In order to assess the risk of immediate toxic effects (RITE), the model of personal impact thresholds is used. In terms of air pollution, this model is defined by the formula in Equation 2:

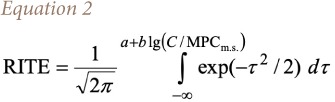
where, *a*, *b* are the parameters depending on toxicological properties of the substance; *C* is the toxicant concentration in the air (mg/m^3^); and MPC_m.s._ is the maximum permissible single concentration of a pollutant in the air. To find RITE, it is necessary to calculate a defined exponential function integral: exp(−τ^2^/2) = e^−*τ*2/2^ (number e = 2,71), where, *τ* is an integration variable that takes a value from −∞ д a + *b* lg(C/MPC_m.s._) and *dτ* is a differential variable *τ*.

The risk of chronic intoxication (RCI) is defined for a certain time period using Equation 3.

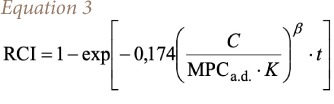
where, *β* is the coefficient that takes into account toxic properties of the substance; *K* is the parameter depending on the class of hazard of the substance; *C* is the pollutant concentration in the air over time (*t*) (mg/m^3^); and MPC_a.d._ is the maximum permissible daily average concentration.

The values of *a*, *b*, *β,* and *K* depend on the substance hazard classification and are given in Table 2.

**Table 2:** Coefficient values of a, b, β and K

**Substance hazard class**	**Substance characteristics**	***a***	***b***	***β***	***K***
1^st^	Extremely hazardous	−9.15	11.66	2.40	7.5
2^nd^	Highly hazardous	−5.51	7.49	1.31	6.0
3^rd^	Moderately hazardous	−2.35	3.73	1.00	4.5
4^th^	Low hazard	−1.41	2.33	0.86	3.0

In order to define the level of air pollution attributed to a certain substance, average concentration values along with the highest values of concentration at a certain period of time are identified.

The level of integral risk from simultaneous impact of all investigated substances was calculated with the formula in Equation 4:

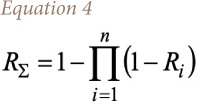
where, *R*_*∑*_ is the level of integral risk from simultaneous impact of all investigated substances; *R*_*i*_ is the health risk value as a result of the impact of *i*-substance; and *n* is the number of substances under investigation.

In Ukraine, according to applicable instructional guidelines of the Ministry of Health of Ukraine, the individual carcinogenic risk, hazard coefficient for exposure to *i*-substance, level of noncarcinogenic risks and hazard index are defined.^7^ The carcinogenic risk was calculated using Equation 5:

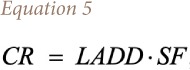
where, *CR* is the carcinogenic risk; *LADD* is the average daily dose over a lifetime (mg/(kg·day)); and *SF* is the slope factor (mg/(kg·day))^−1^.

The average daily dose over a lifetime is defined by Equation 6:

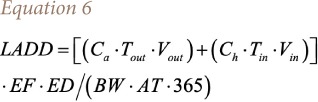
where, *C*_*a*_ is the concentration of the substance in the ambient air (mg/m^3^); *C*_*h*_ is the concentration of the substance in the room air (mg/m^3^); *T*_*out*_ is the time spent outdoors (h/day); *T*_*in*_ is the time spent indoors (h/day); *V*_*out*_ is the breathing rate outdoors (m^3^/hr); *V*_*in*_ is the rate of breathing indoors (m^3^/hr); *EF* is the frequency of exposure (days/year); *ED* is the duration of exposure (years); *BW* is body weight (kg); and *AT* is the exposure average period (years).

The hazard coefficient is defined using Equation 7:

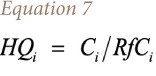
where, HQ_i_ is the hazard coefficient for exposure of *i*-substance; *C*_*i*_ is the level of exposure to *i*-substance (mg/m^3^); and *RfC*_*i*_ is the safe exposure level of *i*-substance (mg/m^3^).

The hazard index is defined using Equation 8:

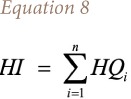
where, *HI* is the hazard index; *HQ*_*i*_ is the hazard coefficients for individual components of the solution of chemical reagents that produce an effect.

The main parameter determining the types of risk considered in this analysis is the concentration of pollutants in atmospheric air, which is measured by the monitoring system or in cases where measurements are absent, modeling is based on data on emissions and meteorological conditions. All other parameters listed in the above formulas are either selected from the relevant reference literature tables, for example Popov *et al.*, for each substance respectively (MPC, slope factor, safe exposure level), or were set in accordance with study conditions.^8^

The authors of the present study have designed a software-modeling complex (SMC) to solve problems of environmental pollution in technogenic loaded territories.^8–10^ The essential advantages of this SMC above other models are the wide range of investigated environmental problems, the presence of a universal database monitoring the sources of pollution and pollutants, POS data, different environmental health values (demographics, population morbidity, etc.) and the existence of a module of environmental risks calculation, which is thought to be the most objective hazard assessment. The SMC database obtained data from different monitoring sources in Ukraine from 2005 to 2017.

The designed SMC makes it possible to observe the spatial distribution of atmospheric air pollution levels and individual risk distributions for a population (risk maps). The risk assessment completes the process of modeling anthropogenic pollution loads on a city's population and defines the criteria for managerial decisions.

**Figure 2:**
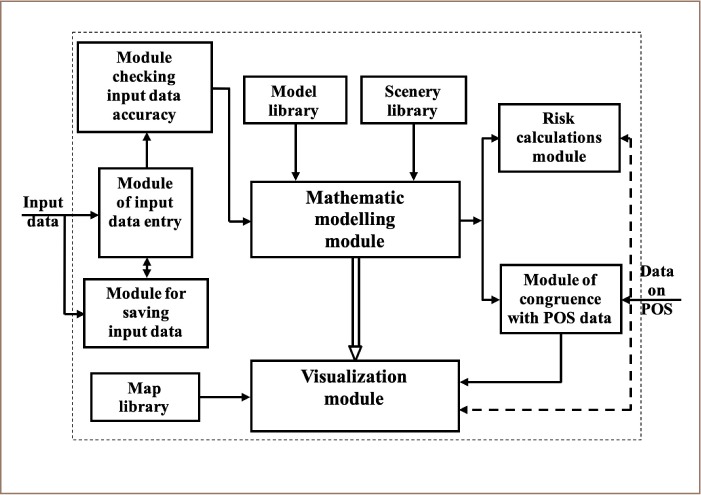
Module structure of risk assessment system

Figure 2 shows the module structure of the risk assessment system that is a part of the SMC. The input modeling data are POS data, pollutant characteristics, sources of pollution parameters, and metrological parameters.

The designed SMC realizes an expert approach to the tasks of risk assessment for public health, corresponding with the current legislation in Ukraine, taking into account regulations of the US Environmental Protection Agency, and grounded by the results of the analysis and appropriate interpretation of ecological monitoring data.^11^,^12^ The given approach includes the following stages:

1. Hazard identification: defining informative parameters (dangerous substances, hazard indices) on the basis of monitoring and expert data.^13^
2. Assessment of the impact of anthropogenic pollution loads as a result of data analysis and spatially distributed modeling: defining and visualizing territories with excess critical concentration of hazardous substances.
3. Assessment dependence, “dose – reaction”: defining with the help of mathematic models the probable risk assessment for carcinogenic and noncarcinogenic diseases.
4. Characterizing risk distribution: risk ranking and taking into account the approved methodology and other means of visual presentation of the obtained results.^7^

The framework of the given approach uses the US Environmental Protection Agency classification of human health risks and reduces uncertainties during the assessment of total noncarcinogenic risk of substances of unidirectional action (*Table 3).*^11^

**Table 3:** Risk Level Classification

**Risk level**	**Hazard coefficient of noncarcinogenic effects for separate combinations**	**Hazard index of noncarcinogenic effects for a group of solutions of unidirectional action**	**Individual lifetime carcinogenic risk**
High	>3	>6	>10^−3^
Alert	1.1–3	3.1–6	1.1·10^−4^–1.0·10^−3^
Acceptable	0.11–1.0	1.1–3.0	1.1·10^−6^–1.0·10^−4^
Minimal (target)	0.1 and less	1.0 and less	10^−6^ and less

## Results

According to the air pollution index, air pollution in the summer months of 2017 was generally characterized as very high. It differed across regions: at POS 7 it was characterized as very high (greater than 14); at POS 5 as high (7–14); at POS 2 as elevated (5–7); and at POS 1 as low (less than 5). In general, the average monthly concentrations of four pollutants in the city exceeded the maximum permissible daily average concentration: formaldehyde by 4.3 times, nitrogen dioxide by 4 times, nitrogen oxide by 1.7 times, phenol by 1.1 times, and suspended substances by 1.3 times. The contents of other substances in the air did not exceed health-based exposure limits.

Since 2007, significant seasonal changes in formaldehyde concentrations have been observed. Thus, in the winter months, this figure does not exceed the MPC, and in the summer the concentration increases sharply and exceeds the MPC by 5–7 times, which is quite dangerous for the most sensitive categories of the city population. These fluctuations may be caused by the intensification of construction and repair work, which is common in the city within each district. However, further investigation is needed to identify all the causes of this situation.

In terms of spatial distribution, the most polluted areas in June were the districts of Bessarabska Square, Oleksandr Dovzhenko street (near Shuliavka subway station), Demiivska Square, Kaunasska street, Obolonsky Avenue, and Peremohy Square (the region of Sviatoshyn subway station), where the pollution level was characterized as very high. Somewhat less polluted, but still within the “high” level were the regions of Semena Skliarenka street, Akademika Strazheska (intercrossing with Vatslava Havela Boulevard), Lesi Ukraiinky Boulevard and Popudrenka street. Pollution levels were lower at Inzhenera Borodina street (area of Darnytsia carriage repair plant) and the Hydropark (area near a subway bridge and highway). A low pollution level was observed at Nauky Avenue (green zone, with high concentrations of parks and forest).

**Figure 3:**
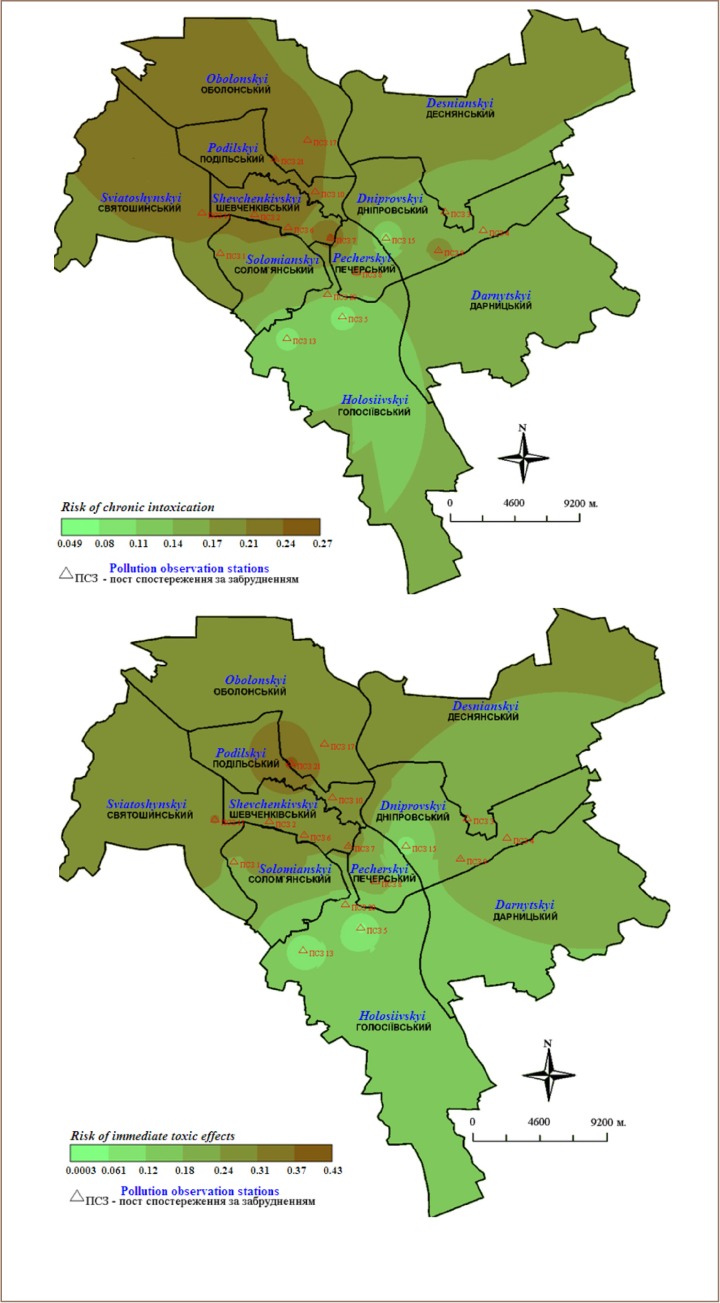
Risk of chronic intoxication (a) and risk of immediate toxic effects (b) as a result of air pollution in Kyiv in January–December 2017

Figure 3 shows map examples of integral RITE and RCI as the result of air pollution in Kyiv during 2017, drawn by the interpolation method of weighted distances. Interpolation was conducted according to the multiplicity of exceeding MPC. The obtained risk values are presented in Figure 3 as outlines in different shades according to the values shown in the map legend.

The risk values for the population of Kyiv proved to be very high. The worst environmental conditions were identified in the northwest where the RCI exceeded 0.2, indicating that more than 20% of the population of the given districts are at risk for chronic diseases. The RITE is also highest for the population in the northwestern part of the city.

The designed SMC makes it possible to obtain the dynamics of risk values for the region under investigation *(Table 4).*

**Table 4:** Dynamics of Risk Values for the Health of the Population in Kyiv

**Location**	**2005**		**2008**		**2011**		**2017**	
	****							
	**RCI**	**RITE**	**RCI**	**RITE**	**RCI**	**RITE**	**RCI**	**RITE**
Hydropark National complex	0.085	0.137	0.063	0.140	0.068	0.025	0.077	0.026
“Ekspocentr Ukraina”	0.061	0.051	0.053	0.120	0.071	0.032	0.087	0.067
Moskovska Square	0.192	0.406	0.060	0.089	0.144	0.157	0.178	0.160
Peremohy Square	0.170	0.458	0.136	0.298	0.210	0.306	0.219	0.226
Bessarabska Square	0.193	0.693	0.275	0.472	0.255	0.321	0.296	0.211
Maidan Nezalezhnosti	0.173	0.514	0.202	0.390	0.212	0.264	0.241	0.202
Damytska Square	0.163	0.386	0.151	0.358	0.174	0.215	0.182	0.187

According to the data, the risk values differ across districts of the city, indicating unstable ecological conditions of atmospheric air in Kyiv. Thus, the lowest risk values were found at the territory of the Hydropark (from 0.025 to 0.137) which is a park complex with minimum impact from traffic and industry.

The greatest risk values are found in Bessarabska Square and Maidan Nezalezhnosti (0.693 and 0.514, respectively, for immediate toxic effects in 2005). In general, the level of RITE in Kyiv is very high, although for some districts it has somewhat lessened compared to previous years and the values of RCI are slowly increasing.

Figure 4a shows the distribution of Kyiv's territory with the risk levels of chronic intoxication according to the avaraged risk data that were obtained from 2011 to 2017. Each bar of Figure 4a represents the percentage of Kyiv's territory (Y axis), which is characterised by the correspondent risk levels (X axis). Figure 4b shows the distribution of Kyiv's territory with the risk of immediate toxic effects according to the averaged risk values for the same period of time (each bar represents part of Kyiv's territory in percentage with the defined risk levels).

**Figure 4a:**
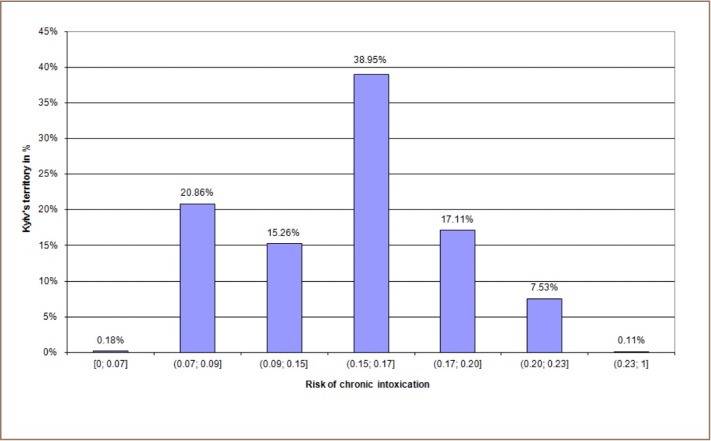
Risk of chronic intoxication in Kyiv territory according to averaged risk values from 2011–2017

**Figure 4b:**
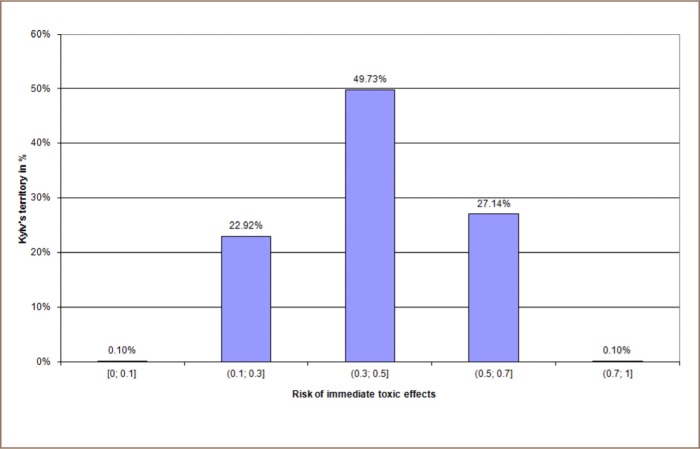
Risk of immediate toxic effects in Kyiv territory according to averaged risk values from 2011–2017

The analysis showed that the majority of Kyiv territory is characterized by 15–17% RCI values, 30–50% RITE values, a hazard index value of 4–6, a hazard coefficient value (for nitrogen dioxide) from 2–2.5, and a carcinogenic risk (for formaldehyde) from 2·10^−5^–5·10^−5^ (*Table 5*).

**Table 5:** Analysis of Kyiv Area Distribution According to Hazard Coefficient and Carcinogenic Risk Values

**Value (risk type and pollutant)**	**Year**			
	**2005**	**2008**	**2011**	**2017**
Hazard coefficient				
Nitrogen dioxide 2–2.5	41%	67%	60%	57%
Carcinogenic risk				
Formaldehyde 2·10^−5^ – 2.5·10^−5^	55%			
Formaldehyde 3·10^−5^ – 4·10^−5^		64%		
Formaldehyde 4·10^−5^ – 5·10^−5^			53%	51%

In order to obtain a more precise environmental risk assessment, it is necessary to modernize the existing monitoring system, increase the number of observation stations and measurements of harmful pollutants. Risk value deviations depend on high concentrations of formaldehyde, which is not measured at some observation stations. The absence of values for some pollutants and the lack of statistical data affect the accuracy of the obtained results and limit the possibilities of their current interpretation.

## Discussion

There have been many studies of the impact of air pollution on population health. Min *et al.* and Mate *et al.* have reported on the risk of mortality from air pollution by solid particles with a diameter of ≤ 10 μm, nitrogen dioxide and sulfur dioxide.^14^,^15^ In Buoli *et al.*, Italian scientists investigated the effect of particulate matter and nitrogen oxides on the mental health of the population, in particular the risk of new symptoms of depression.^16^ In work by Sanidas *et al.*, American scientists stated that air pollution is a significant risk factor for hypertension in all age groups.^17^ Studies have shown that the presence of particulate matter of different sizes in city air increases the risk of cardiovascular disease and significantly increases the likelihood of adverse health effects such as chronic cough, asthma, renal failure and infertility.^18^,^19^ In a study by Yang *et al*., Chinese and American scientists investigated the association between the increase in the number of stillbirths with increasing average daily concentrations of particulate matter with a diameter of ≤2.5 and ≤10 μm at different stages of pregnancy.^20^

The results of the present analysis are generally in agreement with the conclusions of previous investigations. For example, an analysis of macro scale observations from 1997–2013 reported continuous pollution of carcinogenic substances and the variability of their concentration in separate periods in Kyiv's atmosphere air was found to depend on the character of industrial activities.^21^ In addition, the results indicated stable pollutants slightly above the MPC for benz/a/pyrene, formaldehyde and lead. All of the benz/a/pyrene samples exceeded the MPC by 2.3–3.3 times, almost all granules (particles) were 1.1–1.5 times bigger than their normal size.

The above-mentioned study calculated the total carcinogenic risk for human health in Kyiv under the impact of eight hazardous substances (benz/a/pyrene, formaldehyde, N-nitrosodiethylamine, nitrosodiethylamine, cadmium, lead, nickel and chrome VI) from 1997–2013. The total carcinogenic risk deviated within the limits of 1.5–1.9·10^−3^ of the risk level. According to the US Environmental Protection Agency classification, these health risk levels are considered high (>10^−3^) and require reduction measures.^11^ Thus, air pollution levels in Kyiv are not safe for the health of its inhabitants according to an analysis of carcinogenic substances.

The risk distributions and risk dynamics obtained in the present study explain the annual morbidity growth in Kyiv for diseases of the respiratory system, oncology diseases and thyroid cancer.^22^,^23^ The associations between morbidity level attributed to these diseases in Kyiv and air pollution levels have also been confirmed in studies by Chernichenko *et al.* and the US Environmental Protection Agency.^11^,^24^

The power industry is the largest enterprise contributing to the atmospheric air pollution in Kyiv by stationary sources. Since 2012, energy companies, the public joint stock company (PJSC) Kyivenergo and private joint stock company (PrJSC) “Ecostandart” increased their emissions up to 32.8 thousand tons (contributing 79.5% of the total amount of emissions produces by stationary pollution sources in the city).^25^ Precise data concerning the volume of emissions of the largest stationary pollution sources over recent years is unavailable, as this information is not provided on the website of the Ministry of Environment and Natural Resources of Ukraine. However, since 2017, Darnytsya has used coal of higher quality with less ash content and higher caloric content. Thus, the amount of particulate matter emissions in 2017 was reduced by 632.0 tons or by 27.8% compared to 2016.^26^ The largest enterprises in Kyiv that influence the atmospheric air quality are given in Table 6 and their location is shown on the map *(Figure 1).*

**Table 6:** Largest Enterprises Influencing Atmospheric Air Quality in Kyiv(with Reference to Figure 1)

**Number on the map**	**Object**	**Number on the map**	**Object**
1,2	TCPS-5 (pipes 1 and 2)	16	PrJSC “Kyiv Electric Car Repair Plant”
3	TCPS-4 (Darnytska)	17	SE, “State-Run Enterprise of Special Tool Engineering, Arsenal”
4	Korchevatsky building materials mill	18	Chipboards plant “Avers”
5	Plant “Energyia”	19	LLC “Kyivguma”
6	Branch “Damytsia Carriage-Repair Plant” owned by PJSC “Ukrzaliznytsya”	20,21,22	PrJSC Asphalt and concrete plant “Stolychnyi” (pipes 1, 2, 3)
7	Bortnyky aeration station	23	Locomotive depot “Darnytsya”
8	Open joint stock company “Ukrplastyk”	24	Plant “Radykal”
9	TCPS-6 (pipe 1)	25	PrJSC “Kyiv Glass-Container Plant”
10	TCPS-6 (pipe 2)	26	Banknote-printing and minting works of “National Bank of Ukraine”
11	PJSC “Plant Mayak”	27	State joint stock holding company “Artem”
12	SE “Plant 410 of Civil Aviation”	28	PJSC “Pharmak”
13	PJSC Scientific and production center “Borshchahivskyi Chemical and Pharmaceutical Plant”	29	SE “Generator Plant”
14	Specialized utility company “Kyiv Crematorium”	30	PrJSC Plant “Smithy at Rybalskyi”
15	Joint stock company “First Kyiv Machine-Building Plant”		

Abbreviations: TCPS, thermal cogenerating power station; SE, state enterprise; LLC, limited liability company.

According to data on mobile pollution sources by the General Statistics Department, the amount of polluting emissions in Kyiv has been declining (200.7 thousand tons in 2006, 236.4 thousand tons in 2010, 215.8 thousand tons in 2013, and 144.3 thousand tons in 2015). Unfortunately, in 2016 no calculation of polluting emissions by mobile polluting sources was conducted. In addition, emissions of sulfur dioxide (converted to nitrogen dioxide) increased between 2016 and 2017 (*Table 7*).^27^

**Table 7:** Dynamics of Dioxide and Nitrogen Dioxide (Converted to Nitrogen Dioxide) Emissions from Mobile Pollutions Sources

**Year**	**Total thousand tons**		**Sources**				**In the calculation**			

			**Stationary polluting sources**		**Mobile polluting sources**		**Per capita, kg**		**Per 1 km^2^ of the region area, kg**	

	**Sulfur dioxide**	**Nitrogen dioxide**	**Sulfur dioxide**	**Nitrogen dioxide**	**Sulfur dioxide**	**Nitrogen dioxide**	**Sulfur dioxide**	**Nitrogen dioxide**	**Sulfur dioxide**	**Nitrogen dioxide**

2005	5.9	29.5	4.7	13.1	1.2	16.4	2.2	11.1	7.1	35.3
2008	8	32.1	6	8.5	2	23.6	2.9	11.7	9.6	38.4
2011	11.9	31.9	9.9	10.7	2	21.2	4.3	11.4	14.2	38.2
2015	8	22.1	6.5	6.8	1.5	15.3	2.8	7.6	10	26.4
2016	12.3	7.5	12.3	7.5	no data	no data	4.2	2.6	14.8	8.9
2017	15	9.3	15	9.3	no data	no data	5.1	3.2	17.9	11.2

## Conclusions

Analysis of the results of atmospheric air monitoring data demonstrate an unfavorable environmental health situation in Kyiv from 2016–2017. Significant excesses are noted for formaldehyde, nitrogen dioxide, nitrogen oxide, phenol and suspended substances, which significantly raise the risk of toxicity and associated chronic diseases.

Risk assessments, grounded on the basis of monitoring data, reveal that the majority of the city's population is exposed to pollution levels significantly surpassing acceptable limits. The results of the present analysis show that unhealthy atmospheric air conditions have contributed to the increasing morbidity rate in Kiev by 20–30%. The worst situation was observed in the northwestern part of the city.

Further studies are needed examining the status of atmospheric air in Kyiv along with implementation of necessary measures aimed at air quality improvement. From a public health viewpoint, planning and execution of measures aimed at reducing toxic air levels are urgently needed, along with programs designed to inform the public about the influence of pollution on the health of the residents in Kyiv.
